# Audit of Admissions of Older People From a District General Hospital to Psychiatric Units Facilitated by Liaison Psychiatry Services

**DOI:** 10.1192/bjo.2025.10559

**Published:** 2025-06-20

**Authors:** Mandakini Bhowmick, Josie Jenkinson, Vanessa Mabiala, Shazia Kashif

**Affiliations:** Surrey and Borders Partnership NHS Foundation Trust, Leatherhead, United Kingdom

## Abstract

**Aims:** To explore whether locally described ‘gold standards’ have been met for transfers of older people (over 65 years of age) from a district general hospital to inpatient psychiatric units and to generate suggestions for further quality improvement.

Following concerns raised by inpatient consultants about patient safety during transfer of care from local acute trusts to the mental health trusts, gold standards were identified in collaboration with liaison and inpatient psychiatry consultants. Based on these gold standards, a transfer checklist was developed to support psychiatric liaison and acute hospital staff in facilitating transfers. This checklist includes documentation by the acute trust to confirm that the patient is medically optimised prior to transfer, medical and nursing handover to the receiving team, attachment of relevant clinical documentation, including discharge letters and keeping patients and their next-of-kin updated.

**Methods:** We prospectively collected National Health Service (NHS) numbers and dates of transfer for all older people transferred from the district general hospital to inpatient psychiatric units between 1 January2024 to 30 June2024. Data was anonymised, stored in an encrypted file and accessed only on hospital computers. The audit was registered with both the acute and mental health trust. Electronic patient records (EPRs) of both trusts were reviewed retrospectively for evidence of completion of the transfer checklist.

Audit sample: Over the six-month data collection period, 42 older people were transferred from the district general hospital to inpatient psychiatric wards.

**Results:** For more than 90%of the patient transfers, evidence of the following documentation was present: Mental Health Act paperwork and H4 forms (36 out of 36 for whom this was required), patients being informed of transfer (40), discharge letters sent to General Practitioners (40) and confirmation of medical optimisation prior to transfer (38).

Fewer than half of the patients had evidence of National Early Warning Score (NEWS) charts (17), oral intake records (13) and Deprivation of Liberty Safeguards (DOLS) paperwork (4 out of 9 for whom this was required) being sent.

Performance in the remaining areas was variable between 50% (26) to 90% (40).

**Conclusion:** The transfer checklist was adhered across several areas, however key issues were identified, namely NEWS charts, oral intake and DOLS paperwork not having been sent. Audit findings will be presented to key stakeholders at the acute trust. This will be accompanied by teaching to try and improve on these issues followed by a repeat audit. Findings will also be presented to liaison service teams to agree on how to incorporate quality control of transfers into standard operating procedures.

